# Structural analysis of Wss1 protein from saccharomyces cerevisiae

**DOI:** 10.1038/s41598-017-08834-w

**Published:** 2017-08-15

**Authors:** Xiaoyun Yang, Yanhua Li, Zengqiang Gao, Zongqiang Li, Jianhua Xu, Wenjia Wang, Yuhui Dong

**Affiliations:** 1 School of Life Science, University of Science and Technology of China, Hefei, 230026 China; 2 Beijing Synchrotron Radiation Facility, Institute of High Energy Physics, Chinese Academy of Science, Beijing, 100049 China; 3 Key Laboratory of RNA Biology, Institute of Biophysics, Chinese Academy of Sciences, Beijing, 100049 China; 4 School of Science, Qilu University of Technology, Jinan, 250353 China

## Abstract

Wss1 is a DNA-protein crosslinks (DPCs) repair protein, which is responsible for degradation of the protein components in DPCs. In this investigation, crystal structure of the protease domain from *saccharomyces cerevisiae* Wss1 (ScWss1) was solved and was compared with the known crystal structure of *Schizosaccharomyces prombe* Wss1 (SpWss1). It is found that the cleft near zinc ion to be the most conserved core region of Wss1 and that the electronic surface distributions vary greatly between the two homologs. Solution architecture of the full-length ScWss1 was further investigated by small-angle X-ray scattering (SAXS), which indicated the protein contains a flexible region inside. Finally, based on the structural information, a mechanism was proposed about how the enzyme is activated by DNA substrates.

## Introduction

DNA-protein crosslinks (DPCs) arise upon exposure to ionizing radiation, UV light, and are particularly caused by endogenously produced reactive compounds such as formaldehyde^1^. If the DPCs are left unrepaired in cell, transcription as well as DNA unwinding can be inhibited, resulting in genome instability or even cell death^1–4^.

Wss1 is a newly discovered DPCs repair protein. Julian Stingele *et al*. find that yeast mutants lacking Wss1 accumulate DPCs and exhibit gross chromosomal rearrangements. They pointed out that Wss1 contributes to survival of DPC-harboring cells by acting on DPCs proteolytically^5–7^. The current model is that Wss1 breaks down the protein components of DPCs, thereby enabling progression of the replicative helicase. The peptide remnant left that are covalently bound to DNA will still block replicative polymerases, but replication can continue upon recruitment of mutagenic translesion synthesis (TLS) polymerases which are able to replicate even across bulky DNA lesions.

Homologs of the Wss1 proteins were widely found in fungi, plants, and metazoans. The most conserved part of the Wss1 family protein is the protease domain, which is responsible for degradation of the protein component of DPCs. High resolution structures of the protease domain from SpWss1 (SpWss1 (17–151), PDB: 5JIG) and its mutant (SpWss1 (17-151)^112E-Q^, PDB: 5LN5) have been reported by Julian Stingele *et al*. at resolutions of 1.0 and 1.75, respectively^8^. The overall structure and the catalytic center of the protease domain from SpWss1 were described elaborately in this paper. Besides the protease domain, Cdc48/p97, ubiquitin (UBZ), SUMO (SIM) and PCNA (PIP-box) binding motifs and ubiquitin-like domains (UBL) are also typical domains that Wss1 family proteins possess, and no structural information can be found for these domains.

In this investigation, efforts were made to obtain the structure of the full-length Wss1. Function of ScWss1 is better studied than SpWss1. As a result, ScWss1 was chosen for structural investigation in order to be more informative to explain how the structure determines the function. ScWss1 has been proved to be able to cleave itself in a DNA dependent manner. Simple addition of polymeric DNA of different types activated ScWss1 self-cleavage^5^. It is also reported that ScWss1 self-cleavage could be inhibited by EDTA and did not occur using the catalytically inactive variant of ScWss1 (ScWss1^116E-Q^)^5^, so both the wild type protein and the mutant were used for crystallization. However, in our experiments, the two proteins both turned out to be degraded fragments (ScWss1 (21-148) and ScWss1^116E-Q^ (24-149)) in the crystal structures. Therefore, the full-length protein structure was further studied by SAXS. Structural analysis of the high resolution protease domain and the low resolution full-length ScWss1 may provide us new clues to understand the mechanism of the enzyme.

## Results

### Crystal structure of ScWss1^21-148^

Our initial purpose was to crystallize the full-length ScWss1. However, only protease domain (residues from 21-148, abbreviated as ScWss1^21-148^) can be observed in the solved crystal structure. Mass spectroscopy analysis was performed to determine the size of the fragment inside the crystal. As is shown in Fig. S1, the molecule mass of the fragment is about 16 kDa, practically the same as that calculated from the crystal structure (15 kDa), meaning the protein was degraded during the crystallization procedure.

It has been reported that a catalytically inactive variant of ScWss1 (ScWss1^116E-Q^) can significantly reduce self-degradation^5^. So another attempt to obtain the crystal structure of full-length ScWss1 was made to crystalize ScWss1^116E-Q^. This attempt was also unsuccessful, and practically the same fragment (residues from 24-149) was obtained in the crystal. Although the crystal parameters and the crystal shape of ScWss1^21-148^ and ScWss1^116E-Q (24-149)^ vary greatly (Table 1), their structures turned out to be identical with RMSD (Root-mean-square deviation) value of 0.126 Å. The structure of ScWss1^21-148^ was chosen for discussion henceforth, unless otherwise stated.

**Table 1:** Data collection and refinement statistics.

Parameters	ScWss1 (21-148)	ScWss1 116E-Q (24-149)
**Data collection and crystal parameters**		
Wavelength (Å)	0.9793	0.9778
Space group	P6_1_	P6_1_
Cell constants	a = 75.19, b = 75.19, c = 43.74, α = β = 90°, γ = 120°	a = 86.20, b = 86.20, c = 36.29, α = β = 90°, γ = 120°
Resolution	1.76 (1.76–1.79)	1.8 (1.8–1.83)
Number of unique reflections	14078	14258
Completeness (%)	99.9 (100)	99.8 (100)
Redundancy	21.5 (20.5)	9.9 (9.7)
Mean I/σ(I)	82.913 (4.1)	35.975 (3.7)
Molecules in asymmetric unit	1	1
R_merge_ (%)	9.7 (69.2)	6.3 (50.6)
**Structure refinement**		
Resolution range (Å)	1.76–50	1.8–50
R_work_/R_free_ (%)	18.22/21.37	17.32/20.03
Average B-factor (Å^2^)		
Main chain	24.75	27.354
Side chain	29.91	33.544
Waters	34.21	42.886
Ramachandran plot (%)		
Most favored	96.83%	97.58%
Allowed	3.17%	2.42%
Disallowed	0	0
RMSDs		
Bond lengths (Å)	0.007	0.007
Bond angles (°)	1.022	0.833

**Figure 1:**
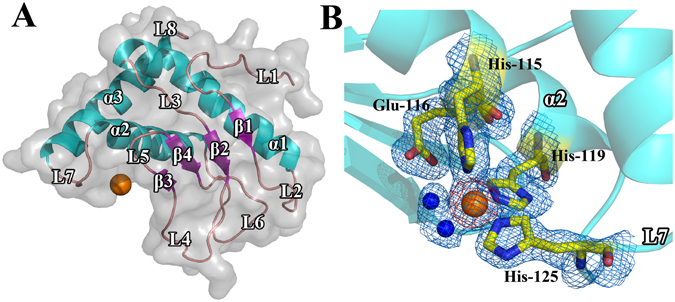
The crystal structure of ScWss1^21-148^ (PDB: 5XBN). (**A**) The overall structure of ScWss1^21-148^ with secondary structure elements labeled. The helices are shown in cyan, sheets in purple, loops in salmon and zinc ion in orange. The molecular surface of the domain is shown in transparent gray. (**B**) Close-up view of the active site, composed of His-115, His-119, His-125, and a zinc ion (orange sphere) in the center, accompanied by Glu-116 as well as a water molecule (blue sphere) between the zinc ion and Glu-116. The 2F_o_-F_c_ electron density map (blue) is contoured to 1σ, whereas the F_o_-F_c_ density map (red) for the Zinc ion is contoured to 3σ.

The overall structure of ScWss1^21-148^ is shown in Fig. 1A. The structure shows a compact globular shape with the maximum diameter of about 50 Å. The protease domain is mainly composed of three α helices and four β strands arranged in a β-α-β-β-β-α-α topology. The top three strands (β1, β2, and β4) are parallel, and the lowermost stand (β3), which creates an ‘upper rim’ to the metal ion is antiparallel to the other β strands. The three α helixes distributed at the back of the β strands and tightly packed into a triangle form.

There is a metal ion inside the crystal and it was proved to be Zinc ion according to the X-ray fluorescence spectrum (Fig. S2). The zinc ion resides at the bottom of the active-site crevice, formed by α2, β3, and loop7. His-115, Glu-116, and His-119, together formed a short zinc-binding consensus sequence HEXXH. The catalytic center of ScWss1^21-148^ is a tetrahedron structure composed of the zinc ion in the center, as well as three histidines (His-125, His-119, and His-115) around (Fig. 1B). His-125 is provided by the loop7, while His-119 and His-115 are provided by the α2 helix. All the three histidines located 2.2 Å away from the Zinc ion. A glutamate residue Glu-116, which was also provided by the α2 helix, located near the Zinc ion (4.8 Å) with a water molecule between it and the zinc ion (Glu-116 - H_2_O; 2.8 Å; H_2_O - Zn: 2.7 Å). Thus, Glu-116 has no direct interaction with the zinc ion. It was probably that the water molecule was polarized by Glu-116 and forms a hydration bond with the zinc ion to perform the catalytic activity together with the three histidines.

**Figure 2:**
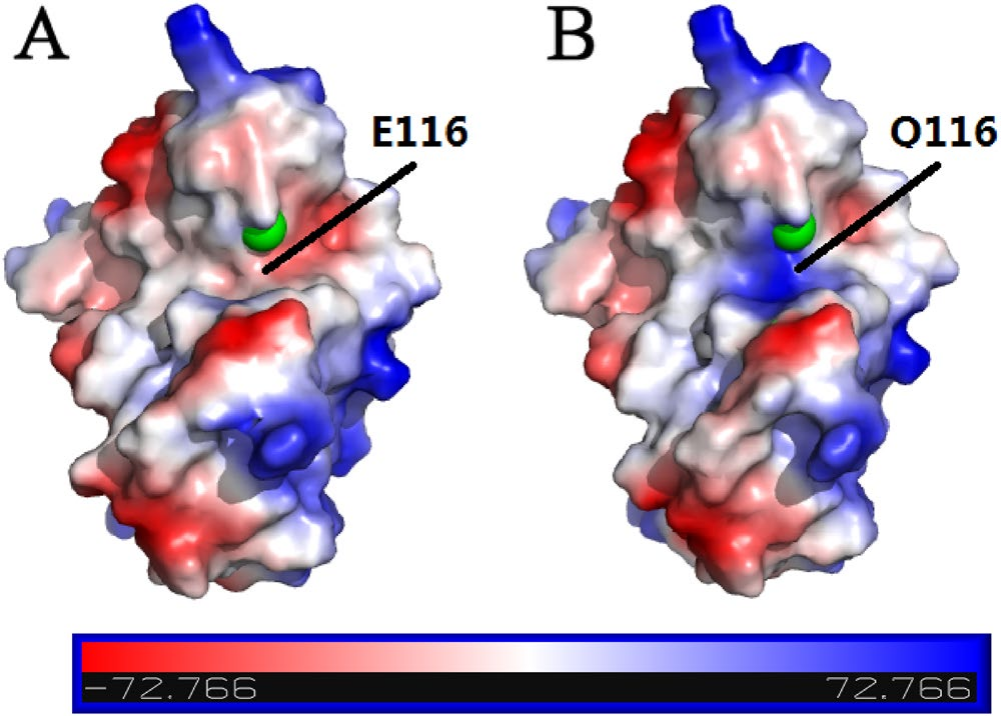
Electronic surface potential comparison between (**A**) native ScWss1^21-148^ (PDB: 5XBN) and (**B**) its mutant ScWss1^116E-Q (24-149)^ (PDB: 5XBV).

The electronic surface distributions of ScWss1^21-148^ and ScWss1^116E-Q (24-149)^ are shown in Fig. 2. Intriguingly, in the structure of the EQ mutant, by changing a negatively charged residue Glu into a neutral residue Gln, the patch became highly positive charged. As it has been confirmed that this mutation does not result in general structural alterations, the significant change in the electric distribution provides an explanation for the prominent reduction of the enzymatic activity.

### Solution structure of full-length ScWss1

**Figure 3:**
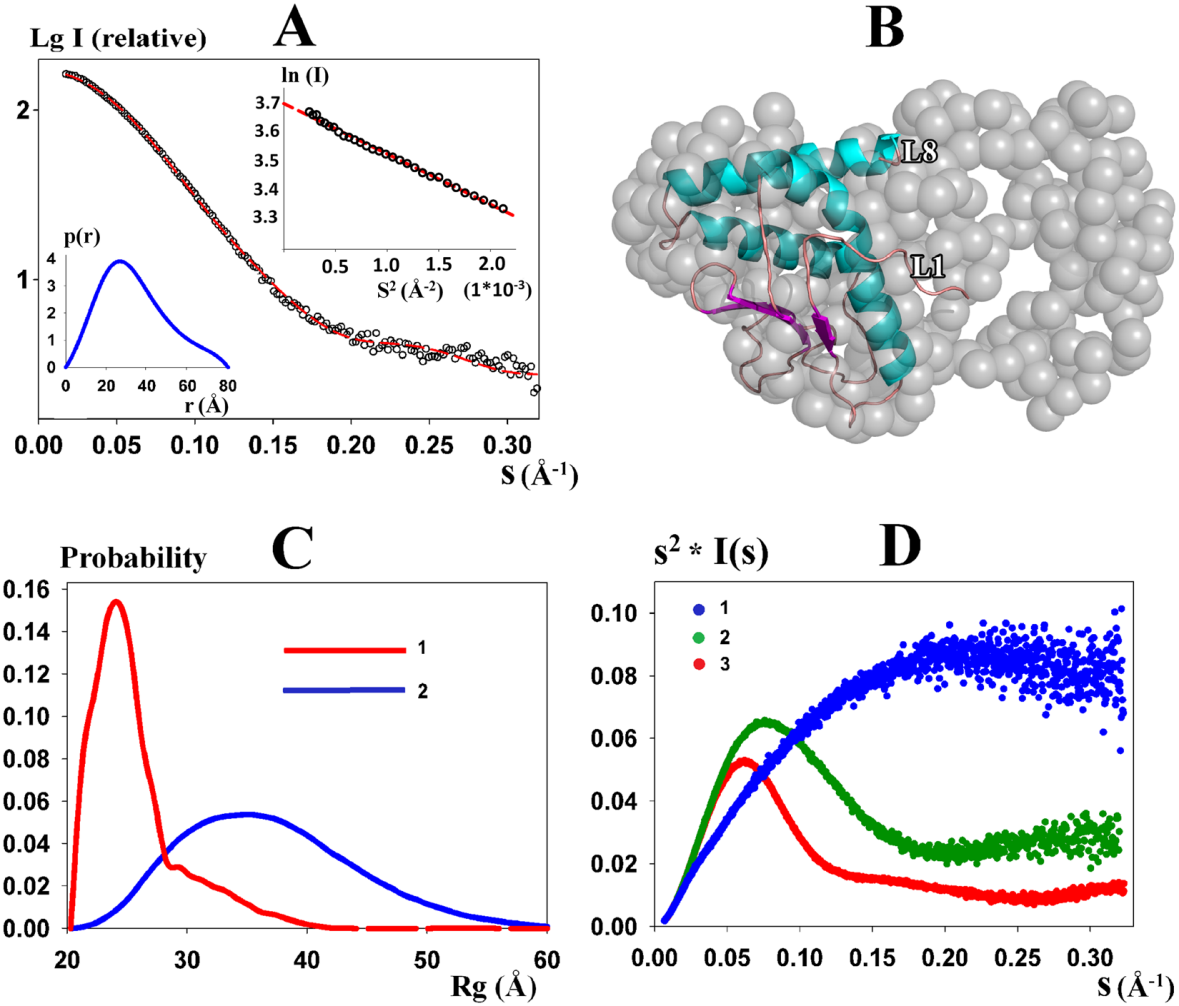
Solution SAXS analysis of the full-length ScWss1 protein. (**A**) Main plot - Experimental scattering data of the full-length protein (black cycles) and the scattering pattern computed from the GASBOR model (red dashes); Insert (left below) - Distance distribution function *p*(*r*) of the full-length ScWss1 protein; Insert (right above) – Guinier plot for the full-length protein. (**B**) Superposition of the GASBOR model and the crystallographic structure of ScWss1^21-148^. (**C**) *Rg* distribution of the optimized ensemble (curve 1) selected from a random pool (curve 2) for the full-length ScWss1 analyzed by program EOM. (**D**) 1 - Kratky plot of a flexible protein (EG5 repeat protein G51-G52); 2 - Kratky plot of the full-length ScWss1; 3 - Kratky plot of a folded protein (BSA).

SAXS experiment was further applied to investigate the structure of full-length ScWss1 in solution. Before SAXS data collection, the purity and homogeneity of the protein was confirmed by dynamic light scattering (DLS, Fig. S3A), which demonstrated the diameter of the protein particle of about 75 Å (Fig. S3B). SAXS profile of the full-length ScWss1 is shown in Fig. 3A. Corresponding structural parameters that are derived from the SAXS data are listed in Table 2. Molecule mass calculated from the SAXS data is 35 kDa, approximately equal to the theoretical molecule mass calculated from the sequence (31 kDa), meaning the full-length protein exists as monomer in solution. Low resolution SAXS model of the full-length protein was further built based on the *p*(*r*) function (Fig. 3A, left below insert) using program GASBOR (Fig. 3B). Ten independent models gave reproducible results (NSD_av_ = 1.25) and demonstrated good approximations to the experimental data with discrepancy value of chi^2^ = 2.5. The final model displayed an ellipsoidal shape for the full-length protein (Fig. 3B), indicating the fragments that are missing in the crystal protruded from the catalytic core into solution, probably for substrates searching and binding activities.

**Table 2:** Overall parameters of the full-length ScWss1 calculated from the SAXS data and the theoretical structural parameters of ScWss1^21-148^ calculated from the crystallography values, including the radius of gyration (*R*_*g*_), molecular mass (*MM*) and maximum diameter (*D*_*max*_).

Sample	ScWss1 (full-length)	ScWss1 (21-148)
R_g_ (Å)	27.7	14.5
MM (kDa)	35	15
D_max_ (Å)	80	50

The part that was invisible or degraded in the crystal was predicted to contain a lot of loops and flexible structures inside. Therefore, we also use an ensemble of conformers to characterize the system. Using the program EOM, a large pool of 10,000 different conformations is generated to analyze the flexibility of the full-length ScWss1, and an optimized ensemble of 50 models that best describes the SAXS data are selected. The selected ensemble of conformations fit the experimental data with chi^2^ = 1.046. The *R*_*g*_ distribution profile calculated from the optimized ensemble contains a main peak at the low *R*_*g*_ region and a dispersion peak at the high *R*_*g*_ region (Fig. 3C, curve 2), implying the full-length ScWss1 mainly exists in a compact state while few of them in a flexible state. Kratky analysis was further made to investigate the flexibility of the protein. The Kratky plot of the full-length ScWss1 located between that of a fully folded protein (BSA, SASBDB code: SASDA32) and that of a total flexible protein (EG5 repeat protein G51-G52, SASBDB code: SASDA37). This result is in consistency with the EOM analysis. Both indicated that the full-length ScWss1 contains flexible regions, which probably serve as connectors between folded protein domains or as docking regions for binding partners.

### Structural comparisons of ScWss1^21-148^ with its homolog SpWss1^17-151^

**Figure 4:**
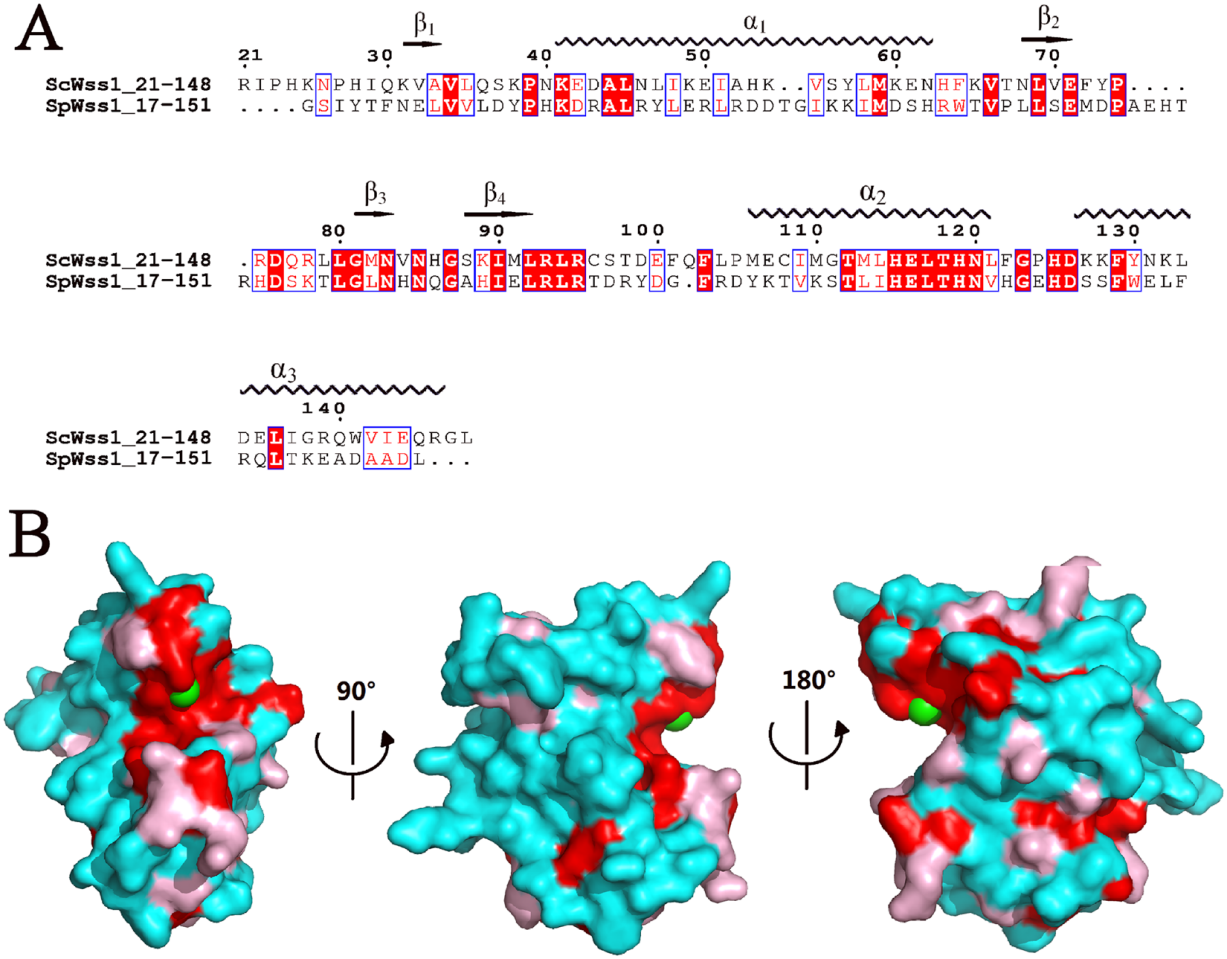
(**A**) Structure based sequence alignment between the protease domain from SpWss1 (PDB: 5JIG) and ScWss1 (PDB: 5XBN), performed using Clustalw (version 2.0) and Espript 3^26^. The conserved residues are boxed in blue. Identical conserved and low conserved residues are highlighted in red background and red letters, respectively. (**B**) Conserved residues are mapped onto the ScWss1^21-148^ surface. Identical conserved residues, low conserved residues, and the most variable residues are shown in red, pink, and cyan, respectively. The zinc ion is shown in green sphere.

A DALI^9^ search for globally similar proteins was performed within the PDB. All hits revealed Z scores <9, except for the crystal structure of the protease domain from S. pombe (SpWss1^17-151^; PDB: 5JIG, Z score =19)^8^. Sequence alignment between the protease domain from SpWss1 and ScWss1 gave a sequence similarity of 28% (Fig. 4A). When the results are mapped onto our structure, the majority of the conserved residues are found to be solvent-exposed (Fig. 4B). Despite the invariant residues are widely distributed throughout the protein, most of them are concentrated in the secondary structural elements. The most significant feature is that the majority of the conserved residues located at the catalytic center around the zinc ion, provided by α2, β3, and loop7_._ Besides the catalytic region, other conserved resides distributed dispersively inside and on the surface of the protein.

**Figure 5:**
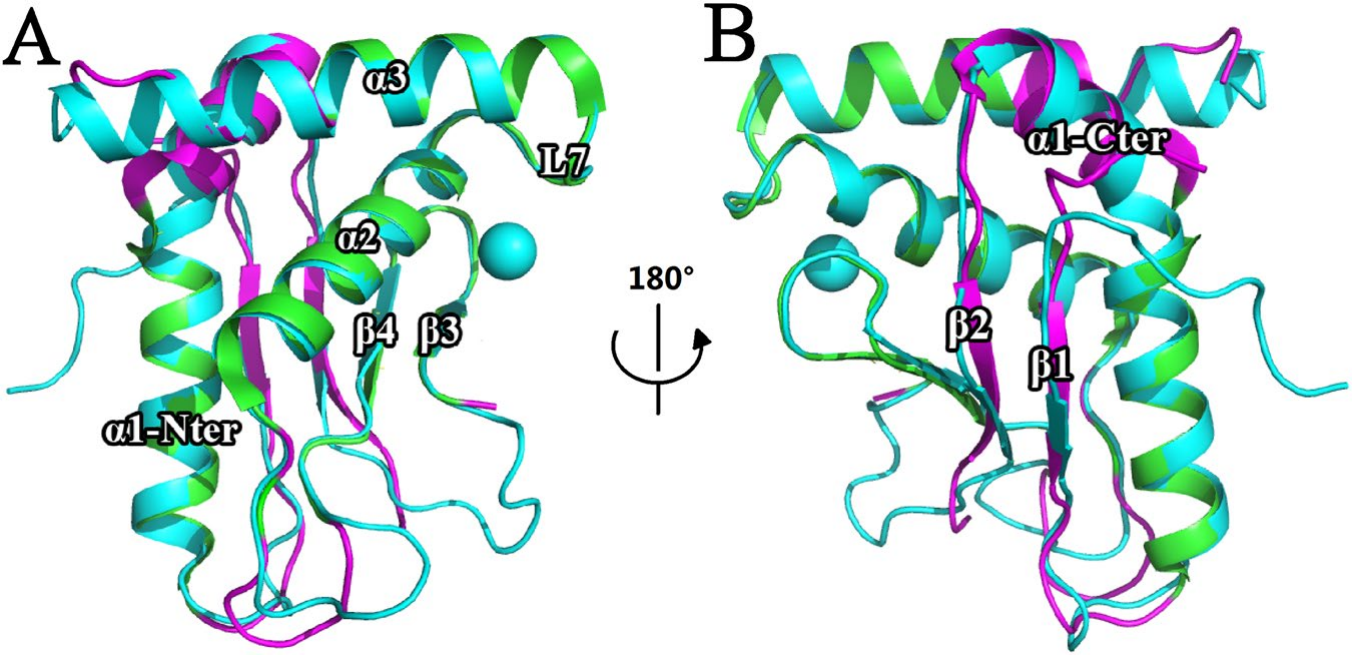
Tertiary structure alignment of ScWss1^21-148^ (PDB: 5XBN) and SpWss1^17-151^ (PDB: 5JIG). The color of ScWss1^21-148^ is shown in cyan. The color of SpWss1^17-151^ is shown in green for the parts which coincide well with ScWss1^21-148^, while for the parts that deviate greater from ScWss1^21-148^ are colored in magenta. (**A**) The parts that coincide well between the two proteins, including α2, α3, β3, β4, loop7, and N terminal of α1. (**B**) The parts that deviate from each other, including β1, β2, the C terminal of α1, and the loops except for loop7.

Structural alignment between ScWss1^21-148^ and SpWss1^17-151^ were further made and their three-dimensional structures were found to resemble each other very much with the RMSD value of 0.473 Å. Especially in the core region around zinc ion (contains α2, α3, β3, β4, and L7), the two structures coincide perfectly with each other (Fig. 5A). One the contrary, for the part which is further away from the core region, the structure is less conserved. β1, β2, the C terminal part of α1, and most of the loops except for loop7 from ScWss1^21-148^ and SpWss1^17-151^ deviated a lot from each other (Fig. 5B). The structural alignment is in consistency with the sequence analysis. Both of them demonstrated the catalytic core to be the most conserved and essential part of the protease domain.

**Figure 6:**
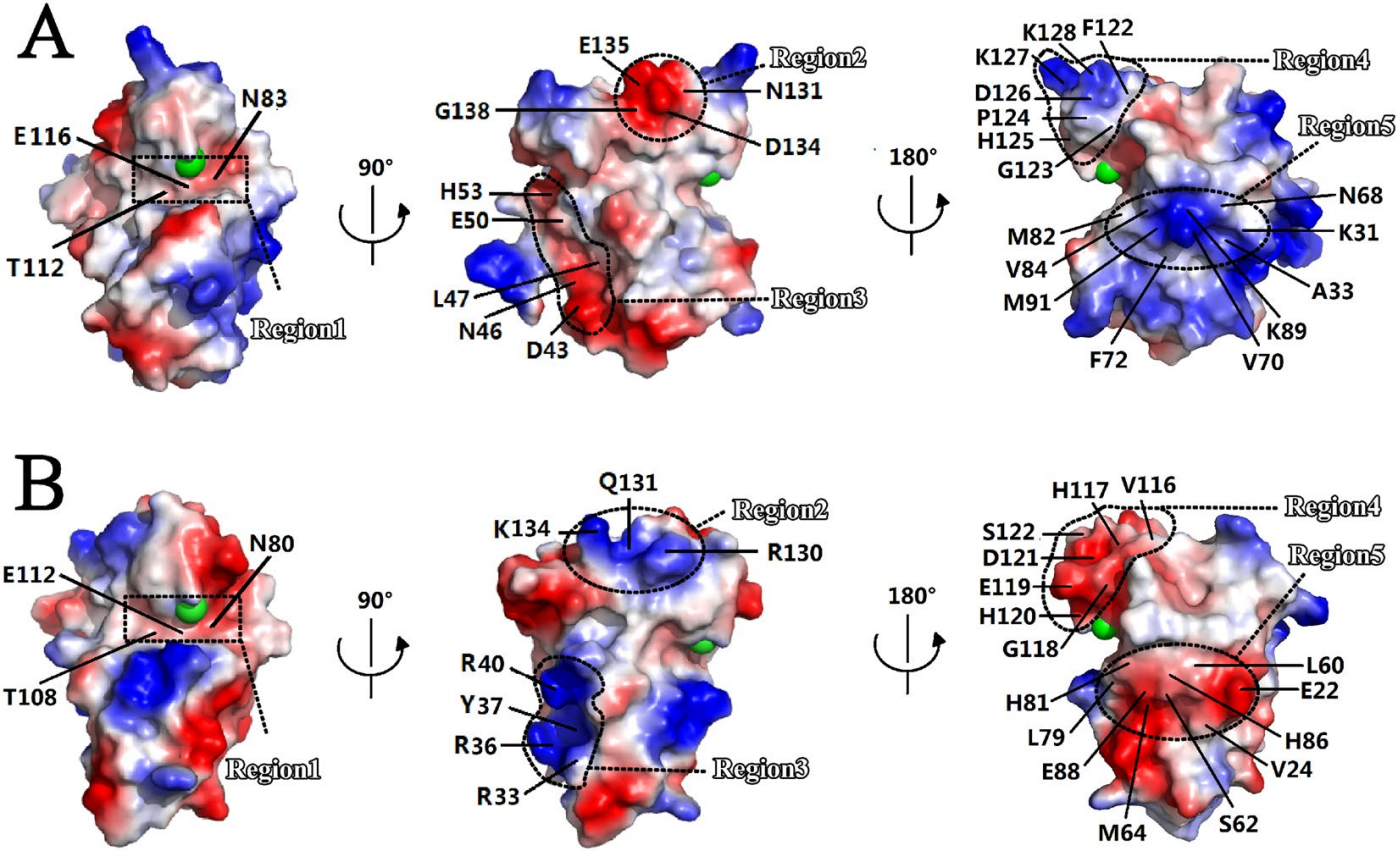
Molecular surface representations of ScWss1^21-148^ (PDB: 5XBN) and SpWss1^17-151^ (PDB: 5JIG) colored by their local electrostatic potential (blue, +7.1KT; red, −7.1KT). (**A**) Electronic surface potential distribution of ScWss1^21-148^ from the side (first panel), front (second panel), and back (third panel) orientations, respectively. (**B**) Electronic surface potential distributions of SpWss1^17-151^ from the same orientations as in Fig. 6A. The zinc ion is shown in green sphere in the figures.

Afterwards, the electronic surface distributions of ScWss1^21-148^ (Fig. 6A) and SpWss1^17-151^ (Fig. 6B) are compared with each other. It has been proved that the patch near the zinc ion is the most conserved part of the protein. In consistent with this point, the electric potential distribution at the patch is also highly conserved. Residues Glu-116 and Thr-112 from α2, Asn-83 from β3 in ScWss1^21-148^ and corresponding residues Glu-112, Thr-108, and Asn-80 in SpWss1^17-151^ formed nearly identical weakly negative charged patch encompassing the zinc ion. (Fig. 6, region1; Fig. S4A and D).

Except for the catalytic core, their electronic surface distributions deviate greatly from each other (Fig. 6, region 2–5), although the two homologs possesses a very high structural similarity (RMSD = 0.473 Å). The front surface of ScWss1^21-148^ is mainly composed of negatively charged residues in region 2 (part of α3, Fig. S4B and E) and region 3 (part of α1, Fig. S4B and E), while the corresponding regions in SpWss1^17-151^ are mainly positively charged. Meanwhile, the electronic surface distributions are nearly opposite for the two homologs in the back surface, especially in region 4 (loop7, Fig. S4C and F) and region 5 (β1, β2, β3, β4, Fig. S4C and F). ScWss1^21-148^ is positively charged in the two regions, while SpWss1^17-151^ is negatively charged in the same place. Thus, the electronic surface distributions are highly unconserved between the two homologs.

## Discussions

In this investigation, structural analysis of the DNA dependent metalloprotease ScWss1 was made for both the crystal structure of the protease domain and solution structure of the full-length protein. The protease domain contains a zinc-binding motif ‘HEXXH’ at the catalytic center, and it was proved to be the most structurally conserved part of the protein. Mutation of a residue (ScWss1^116E-Q^) at the motif can inactive the whole enzyme by changing the surface charge at the patch of the catalytic center, meaning the weakly negative charge in the patch is essential for the protease activity. Another intriguing feature drawn from the crystal structure is that the electronic surface distribution of ScWss1^21-148^ and SpWss1^17-151^ are almost opposite except for the region near zinc ion. The highly unconservative electronic surface distribution together with the absence of an obvious substrate-binding cleft could explain the promiscuity of Wss1 protease with respect to substrate identity.

Notably, self-cleavage occurs during the crystallization procedure for both the native full-length ScWss1 and the mutant ScWss1^116E-Q^. They both degraded into the protease domain, whose molecular mass (15 kDa, confirmed by the mass spectroscopy) equals that obtained from the self-cleavage experiments done by Julian Stingele *et al*.^8^. Considering there might be flexible regions of protein that are invisible inside the structure, the cleavage sites of ScWss1should located in the vicinity of the two terminals of the crystal structure. The crystal structure also confirms the protease domain to be a stable, compact core that is uneasy to be degraded.

**Figure 7:**
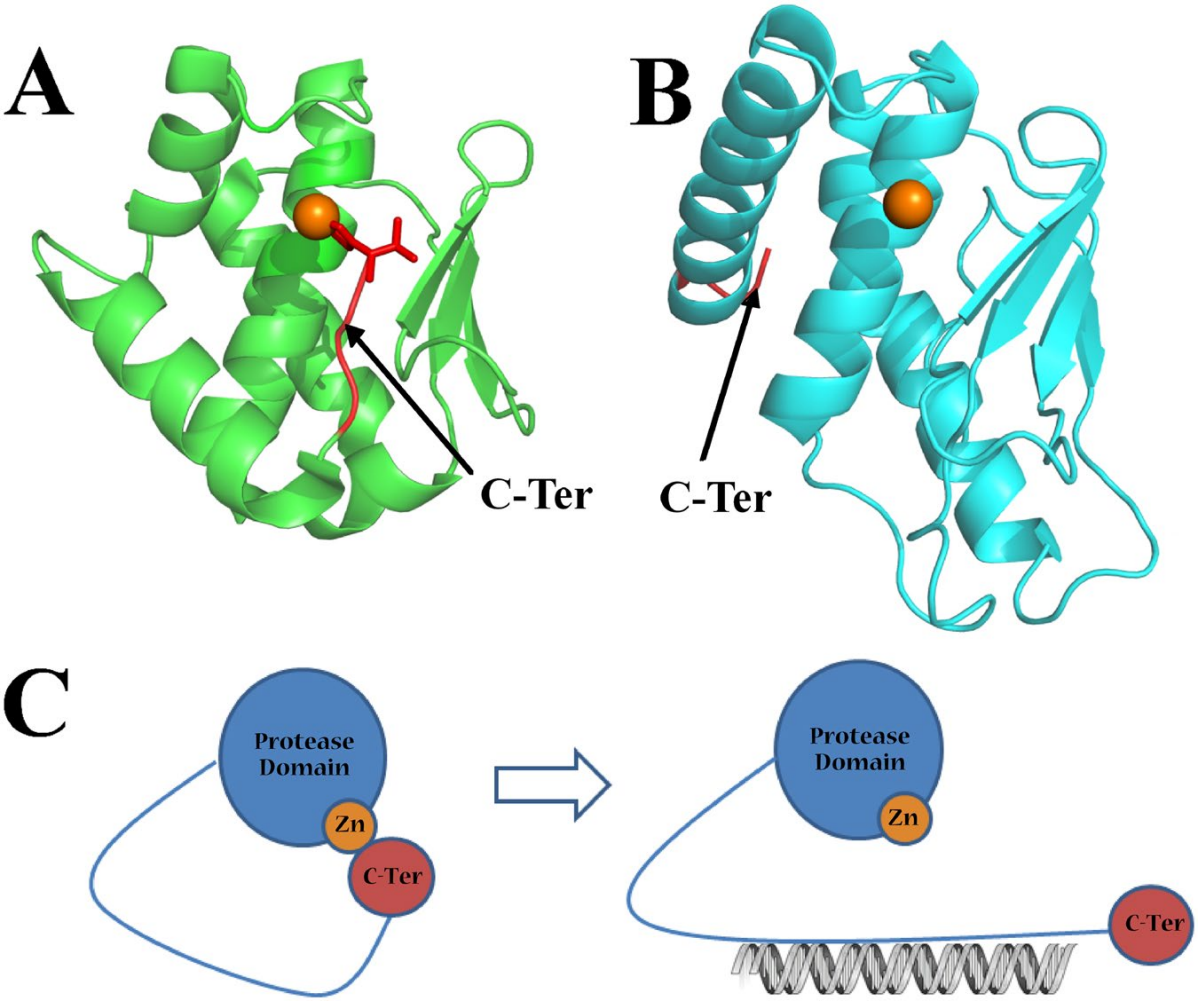
(**A**) Crystal structure of proabylysin (PDB: 4JIU). (**B**) Crystal structure of ScWss1^21-148^ (PDB: 5XBN). The overall structures of the two proteins are shown in cartoon. The C-terminal tails of the two proteins are colored in red. (**C**) Speculated mechanism of ScWss1 activated by the DNA substrates. The protease domain, zinc ion, and the C-terminal domain of ScWss1 were schematically drawn as blue sphere, orange sphere, and red sphere, respectively. The flexible region which links the N-terminal and C-terminal of the protein is drawn as blue line.

Moreover, the crystal structure of ScWss1^21-148^ confirms the model predicted by Balakirev *et al*.^10^ that this protease structurally belongs to minigluzincin family which is first described by Lopez-Pelegrin *et al*.^11^. A structure-based search for ScWss1^21-148^ homologues with DALI program revealed the structure of proabylysin (4JIU) with a DALI Z-score of 6.4, a sequence identity of 13%, and the smallest among non-WLM proteases RMSD value of 2.2. Structural comparison between proabylysin and ScWss1^21-148^ demonstrated the zinc-binding motif ‘HEXXH’ is highly conserved in position, while the other parts of the protein possess greater deviations with each other (Fig. 7A and B). This result is consistent with the structural comparison between ScWss1^21-148^ and SpWss1^17-151^. Both demonstrated the zinc-binding motif to be the most conserved part of the protease domain.

The proabylysin has been proved lacking of hydrolytic activity. Because a C-terminal tail of proabylysin inserted into the active-site cleft and forms interaction with the zinc ion by the residue V105 (Fig. 7A, red part of the protein), thus blocking structural elements essential for catalysis and access of true substrates. Crystal structure of ScWss1^21-148^ is a truncation version of the protein with its C-terminal tail protrudes far away from the catalytic center (Fig. 7B, red part of the protein). It has been proved that Wss1 has no catalytic ability alone, while simple addition of polymeric DNA can activate Wss1 cleavage. Our SAXS results indicated the full-length ScWss1 contains flexible loops inside. Secondary structural prediction demonstrates there is a flexible region located after the C-terminal of the protease domain, and the region was proved to be involved in DNA binding activities^5^. Julian Stingele *et al*. also reported that the DNA-free Wss1 is flexible, and that the addition of DNA increases flexibility significantly^8^. Taken these clues together, we speculated that the protease activity of Wss1 may be inhibited and activated in a similar mechanism as proabylysin. That is to say, when Wss1 is free of DNA, some part at the C-terminal of the protein interacted with the zinc ion and blocks the catalytic center to contact other substrates (Fig. 7C, left), while when Wss1 is in combination with DNA, the flexible region linking the protease domain and the C-terminal part of the protein may be deformed and the obstacle at the catalytic center can be removed, thus the enzyme is activated to be capable of contact and hydrolyze protein substrates (Fig. 7C, right).

## Materials and Methods

Our original research aim was to learn the structure of the full-length ScWss1. However, only catalytic domain was crystallized successfully probably due to self-degradation. As a result, all the cloning, purification, crystallization, SAXS, and dynamic light scattering procedures we mentioned below refer to the full-length ScWss1.

### Cloning

The gene encoding full-length ScWss1 and ScWss1^116E-Q^ were amplified from the *Saccharomyces cerevisiae* S288c genomic DNA. The PCR products were cloned into the pET28at_plus-sumo vector with an N-terminal his-tag prior to the sumo fusion tag and followed by a ULP1 (Ubl-specific protease 1) cleavage site. The plasmid was further isolated and transformed into an *Escherichia coli* BL21 (DE3) star expression strain (Invitrogen).

### Protein purification and crystallization

The cells containing the target proteins (full-length ScWss1 and full-length ScWss1^116E-Q^) were suspended in buffer containing 20 mM Tris pH 7.5, 500 mM NaCl, 1 mM phenylmethylsulfonyl Fluoride (PMSF). After sonication, the lysate was centrifuged at 13000 × g for 50 min. The supernatant containing the soluble ScWss1 and ScWss1^116E-Q^ proteins were purified by affinity chromatography with nickel–nitrilotriacetic acid resin (Bio-Rad) and the 6-his-SUMO tag was removed by overnight hydrolysis with ULP1 protease. The proteins were further purified by Ion Exchange Chromatography (SourceTm15S, GE Healthcare), where the protein was eluted as a single peak at 280 mM NaCl. These fractions were finally purified by gel filtration (Superdex 200, GE Healthcare) pre-equilibrated in buffer containing 20 mM Tris-HCl, pH 7.5, and 150 mM NaCl. The final yield of protein was collected and ultra-filtered to 3.5 mg/ml for crystallization.

ScWss1 and ScWss1^116E-Q^ crystallization screenings were carried out at 293 K using the sitting-drop vapor-diffusion technique. The best crystals of ScWss1 were obtained within two weeks under the condition of 20% (w/v) PEG-3500, 8%v/v Tacsimate, pH 7.0, whereas the best ScWss1^116E-Q^ crystals were grown in buffer containing 10% (w/v) PEG-8000, 20 mM Tris-HCl, pH 7.0, and 0.2 M MgCl_2_ over 10 days. The selenomethionine (SeMet) derivative of ScWss1 was purified and crystallized the same as described above.

### Crystal diffraction data collection, structure determination and refinement

Both wild type and Se-Met substituted single-wavelength anomalous dispersion (SAD) data sets were collected at 100 K on the station BL17U1 of the Shanghai Synchrotron Radiation Facility (SSRF). All data were processed using the program package HKL3000^12^ and collection statistics are summarized in Table 1. The metal ion inside the crystal was confirmed by X-ray fluorescence analysis, while the molecular mass of the fragment inside the crystal was determined by Mass spectroscopy analysis. Selenium atoms were located by the program SOLVE^13^ and the initial phases were used for automatic model building by the program RESOLVE^14^, which produced interpretable electron density. The phases from RESOLVE were transferred into the program ARP/wARP^15^ for further model building. This model was further built and refined against the native data at a resolution of 1.8 Å with the program PHENIX.refine^16^ and COOT^17^. The qualities of the final models were checked with the program MolProbity^18^. Data collection and refinement statistics are given in Table 1. The program PyMOL^19^ was used for preparing structural figures.

### SAXS experiments and data analysis

The full-length ScWss1 was purified as described above. Synchrotron SAXS experiments were performed on the BL19I2 station of SSRF. The scattering was recorded in the range of the momentum transfer 0.018 Å^−1^ < s < 0.321 Å^−1^, where s = 4πsinθ/λ, 2θ is the scattering angle, and λ = 1.5 Å is the X-ray wavelength.

To exclude concentration dependence, three different concentrations, 2 mg/ml, 4 mg/ml, and 6 mg/ml were prepared and measured. No concentration dependence and aggregations were observed during the measurements. All SAXS data were processed with the program package ATSAS^20^. The scattering of buffers were subtracted from that of the samples, and then were extrapolated to zero concentrations using standard procedures and program PRIMUS^21^. Distance distribution function *p*(*r*) was calculated using indirect Fourier transformation and the program GNOM^22^. *Ab initio* low resolution shape restoration was done with the program GASBOR^23^. Ten independent runs for each of the program were compared by the program SUPCOMB^24^ and those with the lowest normalized spatial discrepancy (NSD; a measure of quantitative similarity among sets of three-dimensional points) were chosen as a typical model. Considering the flexibility of the protein, program EOM^25^ was also used to analyze the full-length protein with assemblies of different conformers.

### Dynamic light scattering analysis

The full-length ScWss1 was measured by DLS using DynaPro-MS (ProteinSolutions) at a concentration of 2 mg/ml at 4 °C. 0.1 ml sample solution was centrifuged at 14,000 rpm for 20 min and passed into a 20 ul chamber quartz cuvette. The data were analyzed using the Dynamics 5.0 software.

## Electronic supplementary material

Supplementary Information
