# Supplementary material for: Structural analysis of Wss1 protein from saccharomyces cerevisiae
Source: Sci Rep. 2017 Aug 15;7:8270. doi: 10.1038/s41598-017-08834-w (PMC5557861; doi:10.1038/s41598-017-08834-w)
Supplement: Supplementary file 1 — Supplementary Information [file 41598_2017_8834_MOESM1_ESM.doc]

**Structural analysis of Wss1 protein from** [**saccharomyces**](app:ds:saccharomyces) [**cerevisiae**](app:ds:cerevisiae)

Xiaoyun Yang^1^, Yanhua Li^2^, Zengqiang Gao^2^, Zongqiang Li^3^, Jianhua Xu^2^, Wenjia Wang^4a^, Yuhui Dong^2b^

^1^ School of Life Science, University of Science and Technology of China, Hefei 230026, China.

^2^ Beijing Synchrotron Radiation Facility, Institute of High Energy Physics, Chinese Academy of Science, Beijing 100049, China.

^3^ Key Laboratory of RNA Biology, Institute of Biophysics, Chinese Academy of Sciences, Beijing 100049, China.

^4^ School of Science, Qilu University of Technology, Jinan 250353, China.

^a^ To whom correspondence should be addressed. [Tel: 86-18911035098](mailto:Tel: 86-18911035098); Fax: 86-10-88233201; E-mail: wangwenjia@ihep.ac.cn; postal address: Qilu University of Technology, 3501 DaxueLu, Changqing District, Jinan, 250353, China.

^b^ To whom correspondence should be addressed: [Tel: 86-10-8](tel:86-010-62759743)8233090; Fax: 86-10-88233201; E-mail: dongyh@ihep.ac.cn; postal address: Institute of High Energy Physics, Chinese Academy of Sciences, 19B YuquanLu, Shijingshan District, Beijing, 100049, China.

**
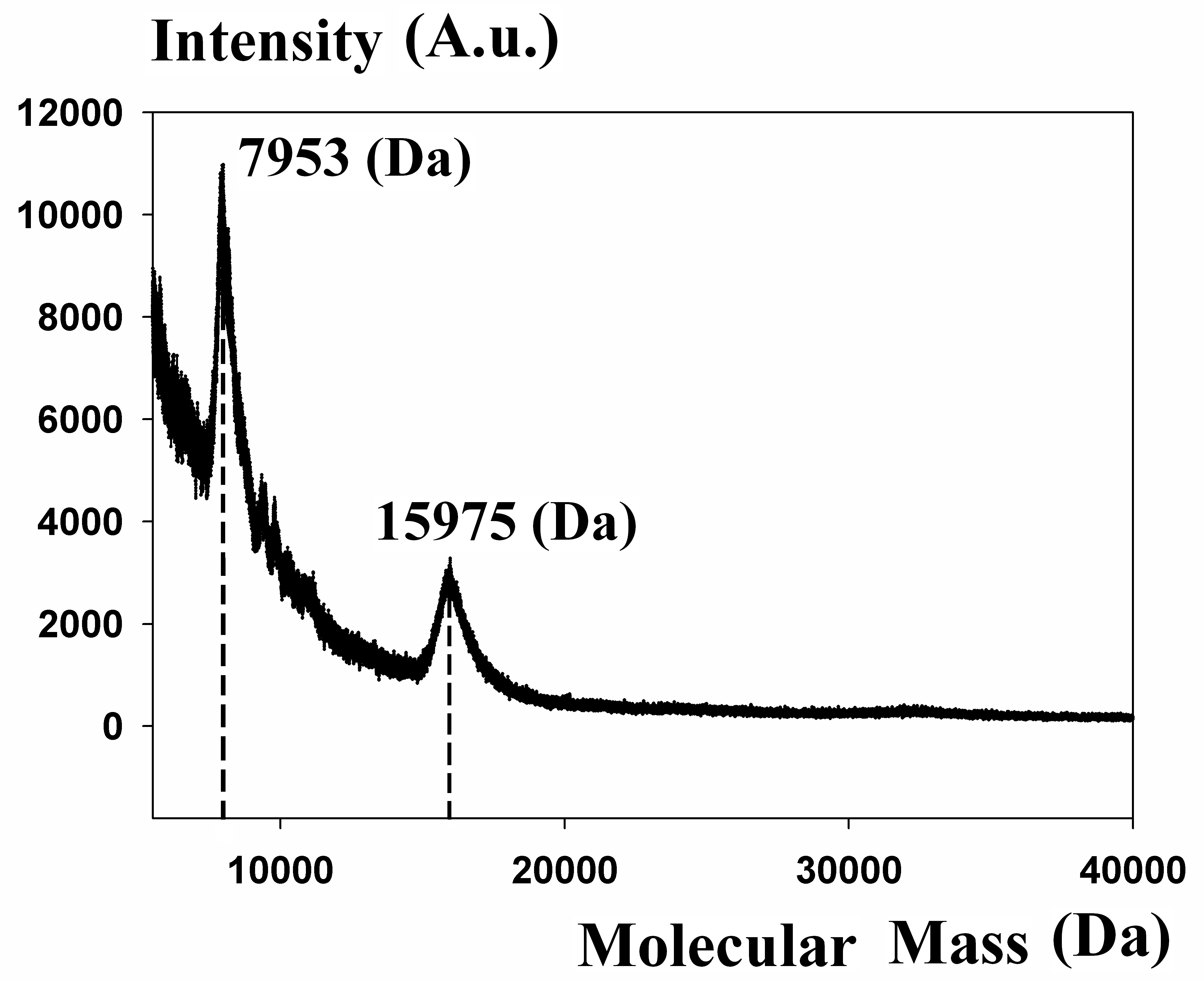
**

**Fig. S1.** Mass spectroscopy analysis of the ScWss1 fragment from the crystal.

**
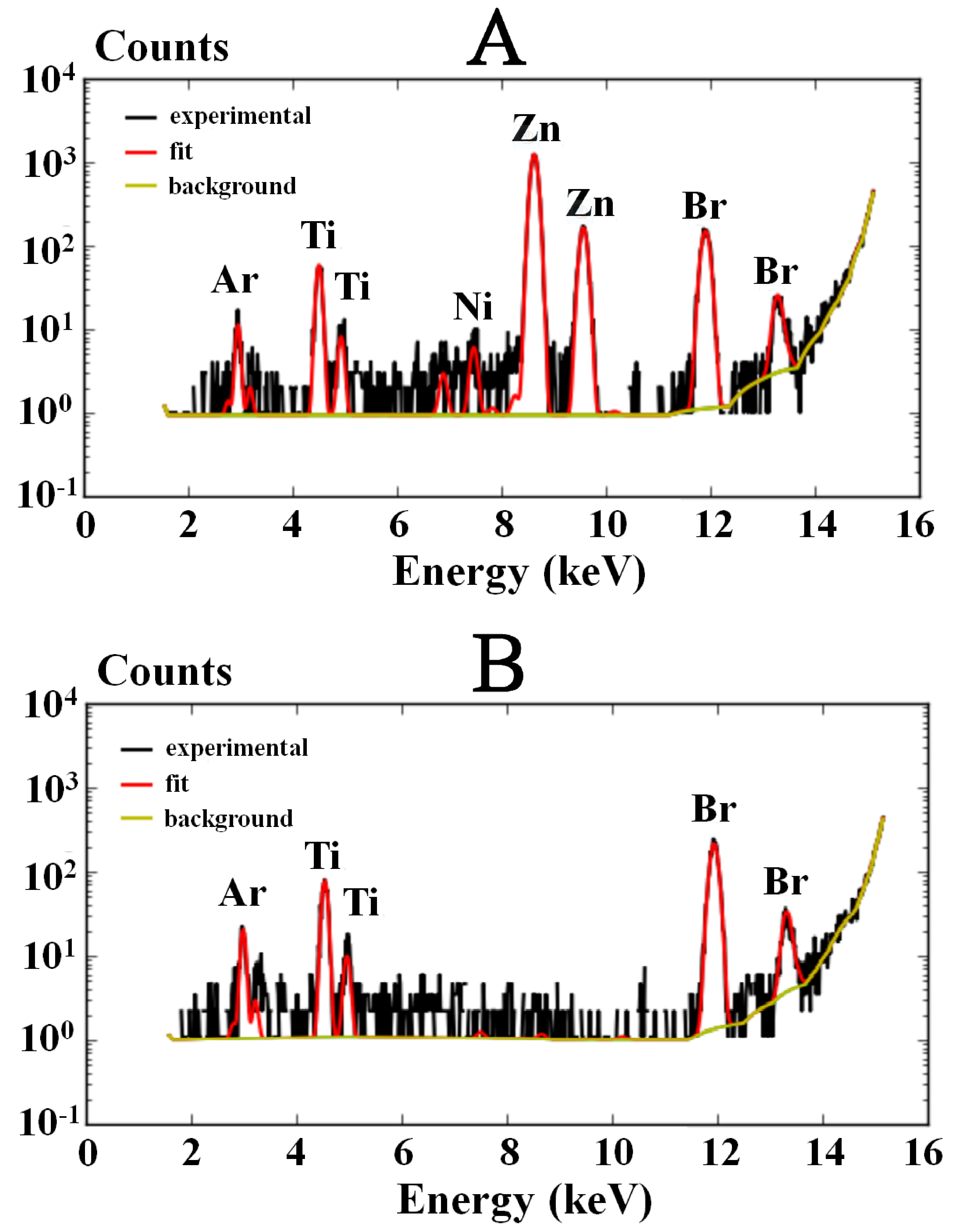
**

**Fig. S2.** X-ray fluorescence spectrum analysis to test the metal ion inside the crystal. The measurement was done with a ScWss1 crystal sticks to a tape. (A) X-ray fluorescence spectrum from the crystal and the tape. (B) X-ray fluorescence spectrum from the tape alone.

**
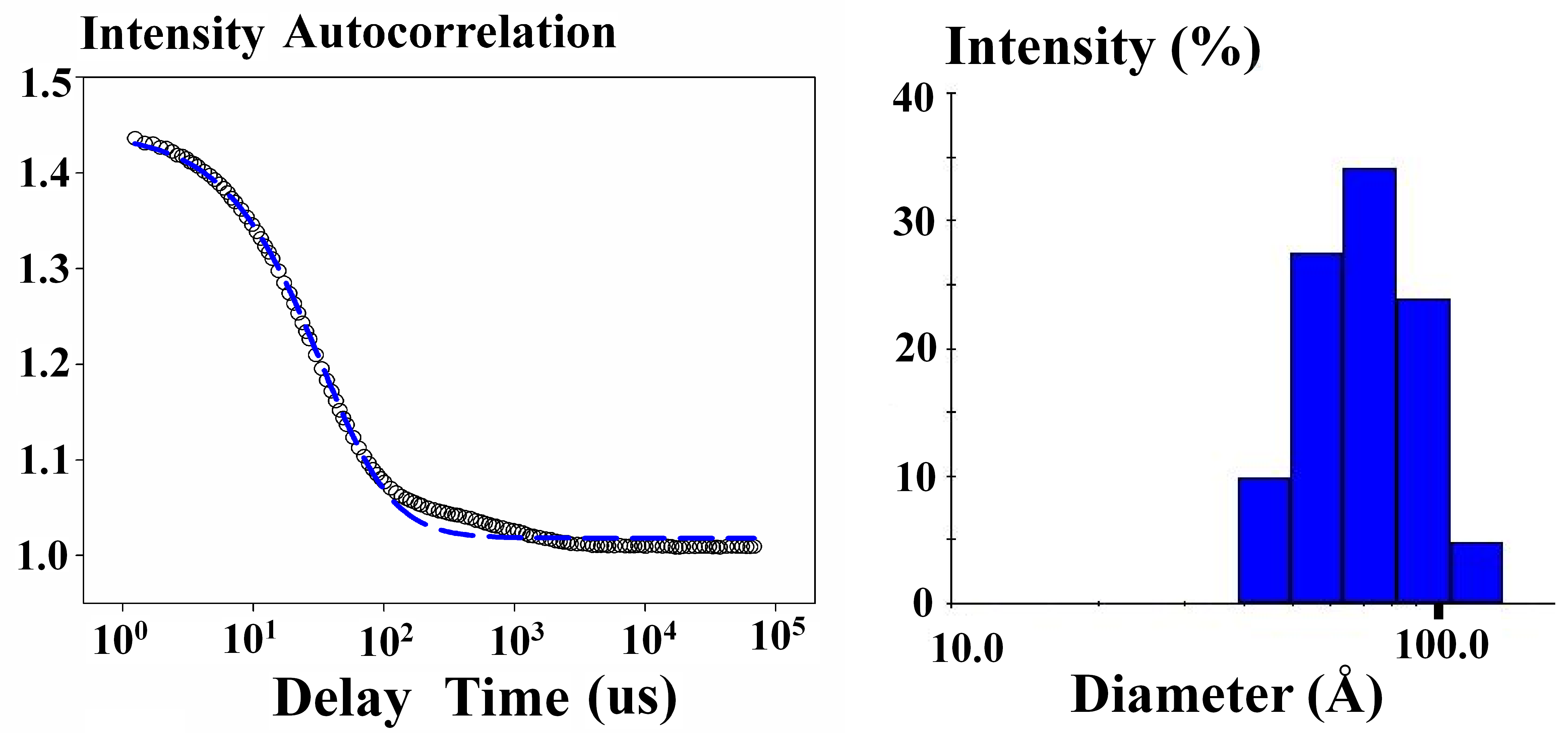
**

**Fig. S3.** Dynamic light scattering analysis of the full-length ScWss1. (A) DLS intensity autocorrelation function. (B) Size distribution of the full-length ScWss1 from DLS.


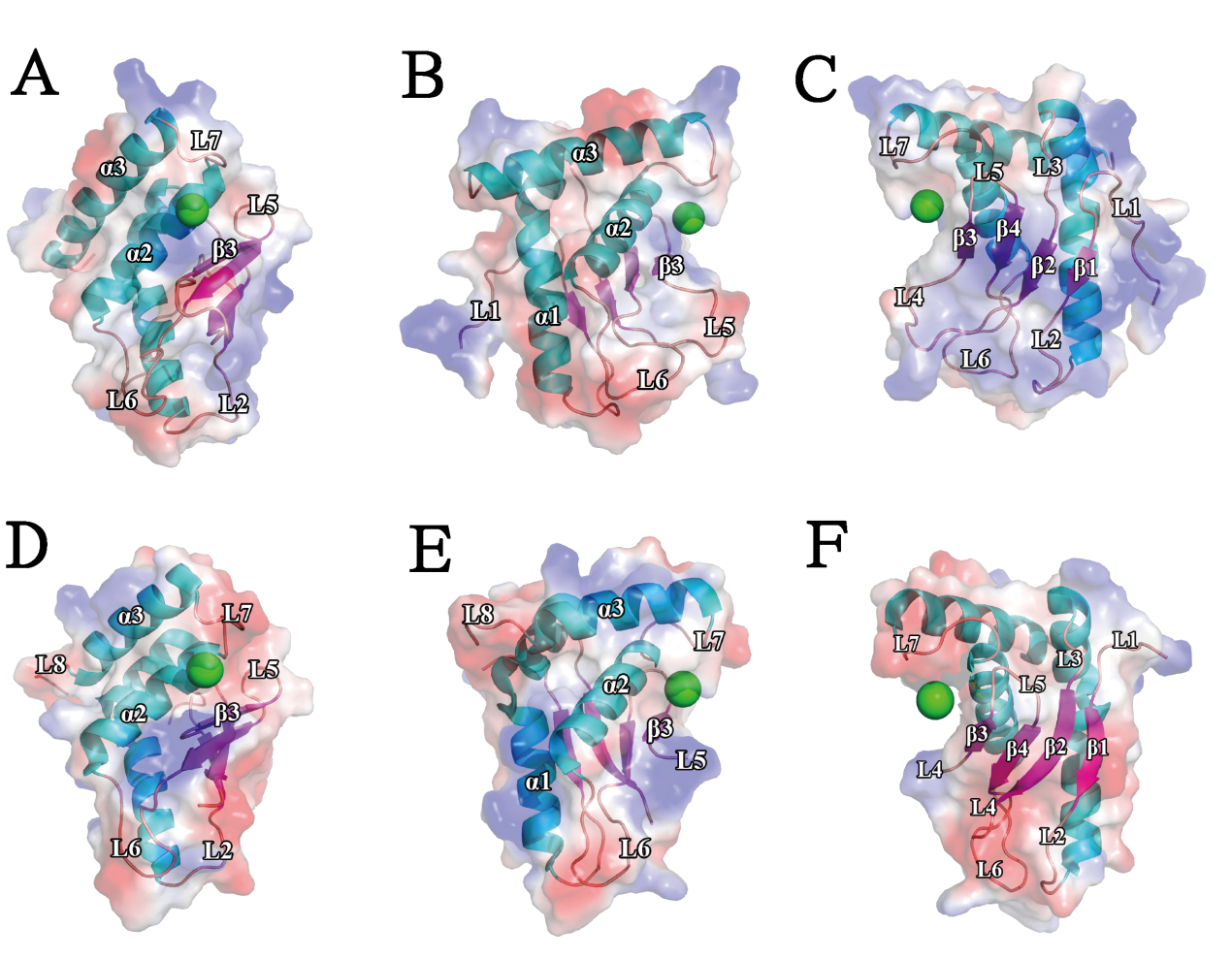


**Fig. S4.** Superposition of the electronic surface distribution with the crystal structure of ScWss1^21-148^ (A - C) and SpWss1^17-151^ (D - F).
